# High Physical Self-Concept Benefits on School Adjustment of Korean Student-Athletes

**DOI:** 10.3390/ijerph17082653

**Published:** 2020-04-13

**Authors:** Young-Jae Kim, Jin-Hoon Jang, Jeong-Hyung Cho

**Affiliations:** 1 Department of Physical Education of Chung-Ang University, Seoul 06974, Korea; yjkim@cau.ac.kr; 2 Sanghyun Middle School, Seoul 06974, Korea; hoon8775@sen.go.kr

**Keywords:** school life, self-concept, youth, athletes

## Abstract

Successful adjustment of student-athletes to their school is an internationally relevant issue. In Korea, school-athletes abandon their athletic activity at a rate of over 40%, suggesting an urgent need to develop measures that allow them to balance sports and academic life. Therefore, this study aimed to investigate the effect of physical self-concept on school adaptation among student-athletes. We analyzed data from 589 student-athletes, including sex and award-winning career as covariates. Then, reliability and validity of scales were obtained. The results showed that student-athletes with higher physical self-concept are more likely to be successful in school adjustment. The effects of physical self-concept on school adjustment were proven to be mediated by sex and award-winning career of student-athletes. This result provides the basis for the importance of recognizing the concept of physical self as a way for student athletes to adapt well to school life. As differences depending on gender and award experience exist, they should be taken into account when teaching student athletes.

## 1. Introduction

Properly maintaining and adjusting to school life is very important during adolescence [1,2,3,4]. The World Health Organization (WHO) recommends daily physical activity for more than 60 min for teens aged 15 to 17. This is because teenagers’ participation in physical activities will help them adapt to school life [5]. However, student-athletes have adjustment difficulties when their activities exceed the WHO-recommended amount [6,7]. Among Korean students, the general dropout rate is less than 1%, but when considering only student-athletes, over 40% abandon athletic activities before graduation. When including those who do not enter professional sports post-graduation, the abandonment rate reaches nearly 90% [8]. These student-athletes also cannot adjust to school life, resulting in >50% transference rate and 11% dropout rate. Furthermore, over half (51.6%) the students stop sports without any external extenuating circumstances [9]. Student-athletes may have more difficulty in adjusting because of several intrinsic characteristics that should be considered when developing assistance programs [10]. As the curriculum in Korea consists of 6 years of elementary school, 3 years of junior high school, and 3 years of high school, it is necessary to adapt to the new environment whenever the class changes. Student athletes in particular have less time to socialize with their classmates due to the inclusion of morning training programs before the first class, after-school training programs, and special training during the competition season. Psychological factors affect girls more than boys [11], while sex-based physical differences also influence adjustment capacity [12]. Additionally, student-athlete success is highly dependent on sporting achievements, and excellent performance in competitions [13] and winning awards increases their likelihood of pursuing a sporting career post-graduation [14]. Taken together, these findings indicate that gender and career awards won should be considered when investigating student-athlete adjustment to school.

Only one study is available on Korean student-athletes [15], but it has a major limitation because participation guarantees the right to study. Therefore, voluntary compensation factors such as academic achievement are not considered despite being an important psychological factor affecting school adjustment [16]. Psychological factors are more effective than behavioral factors at inducing school adjustment [17] and, therefore, should be considered for student-athletes.

High self-concept (positive collection of beliefs about oneself) is linked to better academic and social achievement [18] because it is a variable that affects mental state, psychological well-being, and behavior in adolescents [4,19]. Psychological variables are based on self-assessment. A person with a high self-concept is highly accomplished in academic or social life [20]. Research has found that self-concepts are important factors in school life that affect mental state, psychological well-being, and human behavior [21]. However, the measurement factor of the existing self-concept was developed for general students, so it is difficult to apply it to student athletes who have a different culture from general students [22,23]. Therefore, it is necessary to identify and apply physical self-concept factors appropriate to the characteristics of student athletes. This is because athletes have a better understanding of the body than others to achieve the best performance [23]. Another caveat to consider is that due to differences in curriculum and school life culture, self-concept scales developed in other countries may not transfer to Korea, [24]. Therefore, in this study, we intend to examine the effect of physical self-concept criteria, which has been validated in Korea, on students’ adjustment to school life according to the characteristics of students (gender and award experience).

## 2. Materials and Methods

### 2.1. Participants

This cross-sectional study involved 633 student-athletes (ages 13–18) in Seoul, attending 10 middle and high schools that are part of Korea’s compulsory educational system. All participants were informed via written communication, and parents and legal guardians provided informed consent for student participation. Participants were recruited using the convenience sampling method, and only voluntary participants were enrolled. The final analysis included 589 valid samples after excluding 44 questionnaires with incomplete answers or missing data.

### 2.2. Measurement

#### 2.2.1. Physical Self-Concept

Data were collected using the physical self-concept (PSC) questionnaire [23], adjusted for Korean adolescents from an original developed for English-speaking students [25]. This measurement tool consists of perceived sports ability (SCO) that is part of physical ability, health promotion (HP) that recognizes health state through physical activity and physical activity (PA) that recognizes the body through physical activity. This means that the higher the score, the higher the recognition of your body.

#### 2.2.2. School Adjustment

Data were collected using a 23-item questionnaire [1] adjusted for Korean students from an English-language original. This measurement tool consists measurements of a teacher-to-student relationship (TRE), a relationship with schoolmates (SRE), an adaptation to school class (SCL), and an adherence to school rules (SRU) to measure performance. This means that the higher the score, the better you are adjusting to school life.

### 2.3. Data Analysis

**Figure 1:**
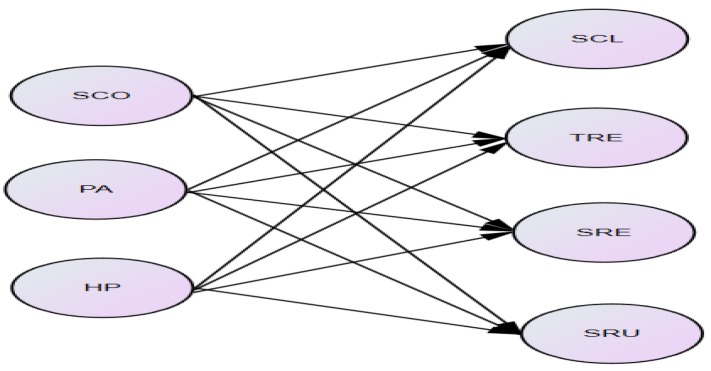
Variables in multiple regression. Independent variables: sports competence (SCO), physical activity (PA), health promotion (HP); dependent variables: school class (SCL), teacher relationship (TRE), schoolmate relationship (SRE), school rules (SRU).

In order to achieve the purpose of this study, the collected data was subjected to statistical analysis using the SPSS program version 25 through the coding process and data cleaning process. The demographic characteristics of the study subjects were analyzed by descriptive analysis and frequency analysis. In order to verify the reliability and validity of the measurement tools, Cronbach’s α, exploratory factor analysis (EFA), and confirmatory factor analysis (CFA) were conducted. Lastly, correlation analysis and multiple regression analysis were conducted to investigate the effects of physical self-concept on school life adaptation. The independent variables were SCO, PA, and HP, while the dependent variables were SCL, TRE, SRE, and SRU (Figure 1).

## 3. Results

Table 1 shows the results of the factorial analysis to confirm the validity of physical self-concept. Participants responded using a five-point Likert scale (“1 = Not at all” to “5 = Very true”). A high total score indicates high physical self-concept. The questionnaire was both reliable and valid (SCO, α = 0.873; HP, α = 0.846, PA, α = 0.850). Table 1. In addition, feasibility has been ensured through exploratory factor analysis and verbatim factor analysis, all of which exceed the standard values.

Table 2 shows the results of the factorial analysis to confirm the validity of School Adjustment Scale. Participants responded using a five-point Likert scale (“1 = Not at all” to “5 = very true”). A high score indicates better school life adjustment. The questionnaire was reliable and valid (TRE, α = 0.806; SRE, α = 0.792, SCL, α = 0.839, SRU, α = 0.872). Table 2. In addition, both exploratory and ascertained factors analysis have met the model adequacy values.

The demographic characteristics of this study are as follows. Male participants (*n* = 372; 63.2%) outnumbered the female participants (*n* = 217; 36.8%) 155 men participated, 1.3 times more than women. Additionally, 47% (278) were middle school students and 52.8% (311) were high school students (Table 3). More student-athletes did not win awards in their career (*n* = 329; 55.9%) than those who did (*n* = 260; 44.1%). For physical self-concept, students scored highest on PA (M = 4.36, SD = 0.618), then HP (M = 4.06, SD = 0.686), and finally SCO (M = 3.84, SD = 0.727). For school adjustment, they scored highest on SRE (M = 4.00, SD = 0.553) and lowest on SRU (M = 3.56).

Table 4 shows the average score of school adaptation according to the concept of physical self, and there found a statistically significant difference. Student athletes who best adapted to school classes were those with high physical activity (M = 3.64, SD = 0.403; F = 9.049, *p* = 0.001). Student athletes with the best relationship with teachers were those with a high level of perception on health promotion (M = 6.37, SD = 0.325; F = 19.636, *p* = 0.001). Students with good friendship with schoolmates were also the groups with high levels of awareness of health promotion (M = 6.04, SD = 0.353; F = 17.130, *p* = 0.001). Finally, the group that adheres well to school rules was found to have a higher awareness of health promotion (M = 3.84, SD = 0.269; F = 14.252, *p* = 0.001).

Table 5 shows the correlation between factors. All physical self-concept and school adjustment variables positively correlated, with the strongest relationship being between SCO and PA (r = 0.643, *p* < 0.001) and the weakest, between HP and SCL (r = 0.224, *p* < 0.001). Multicollinearity was not an issue because no correlation coefficients were over 0.800.

**Figure 2:**
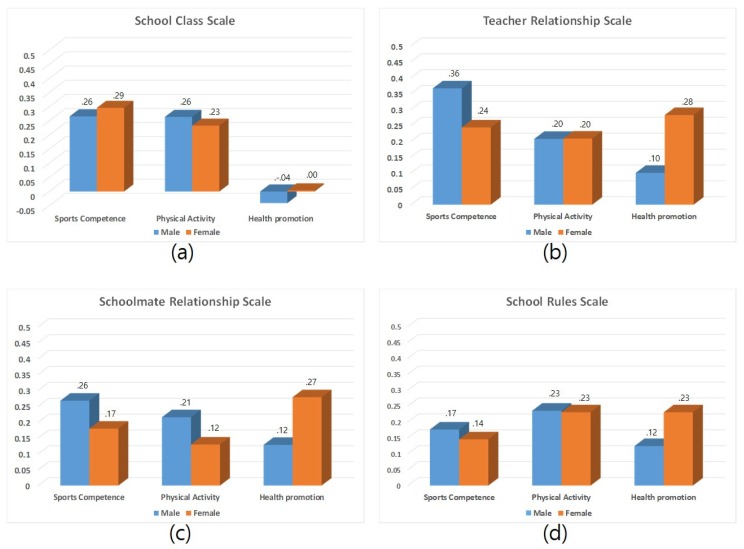
Effect of physical self-concept on school adjustment by gender: (**a**) school class differences, (**b**) teacher relationship differences, (**c**) schoolmate relationship differences, (**d**) school rules differences.

Linear regression revealed that among girls, higher SCL corresponds to SCO (β = 0.296, *p* < 0.001), while higher SCL corresponds to HP (β = 0.233, *p* < 0.001), and higher TRE corresponds to PA (β = 0.282, *p* < 0.001) respectively. Among boys, higher TRE corresponds to SCO (β = 0.366, *p* < 0.001), while higher SCL corresponds to PH (β = 0.264, *p* < 0.001), and higher SRE corresponds to PA (β = 0.128, *p* < 0.001) respectively (Table 6). Figure 2 shows the effect of physical self-concepts on school adaptation based on sex.

**Figure 3:**
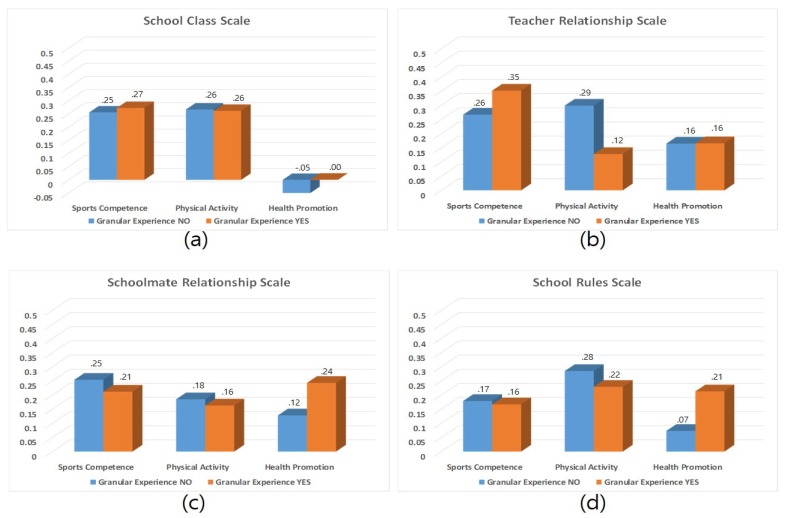
Effect of physical self-concept on school adjustment by award-winning career: (**a**) school class differences, (**b**) teacher relationship differences, (**c**) schoolmates relationship differences, (**d**) school rules differences.

Among students with an award-winning career, higher TRE corresponds to SCO (β = 0.352, *p* < 0.001), while higher SCL corresponds to HP (β = 0.262, *p* < 0.001), and higher SRE corresponds to PA (β = 0.243, *p* < 0.001) respectively. Among those who did not win awards, higher TRE corresponds to SCO (β = 0.267, *p* < 0.001), while higher TRE corresponds to HP (β = 0.299, *p* < 0.001), and higher TRE corresponds to PA (β = 0.165, *p* < 0.001) respectively (Table 7). Figure 3 shows the effect of physical self-concepts on school adaptation based on award-winning career.

## 4. Discussion

This study verified the effect of physical self-concept on the adaptation of school life in student athletes through gender and award experience. However, this sample was recruited by expedient sampling, so you should be careful about the dissection.

Higher SCO is correlated with higher TRE. High-performing players have a good relationship with coaches and their parents. This is more evident in younger players [26,27]. Relationships with others are built on trust. In other words, because the goal of both players and coaches is to achieve good results, high-achieving student players have a lot of experience in winning, which gives them confidence in their coaches, which has the same effect on others who lead them [20]. Higher SCO was correlated with higher TRE, consistent with previous findings [28] indicating that life-skill coaching benefits sports participation. Similarly, another study demonstrated that student-athletes perform better when they communicate constantly with and seek direction from their managers [29]. Thus, we can conclude that a good relationship with coaches could allow student-athletes to form similar bonds with teachers, benefiting school life adjustment.

Analysis of the correlation between physical self-concept and school life adaptation showed that the high physical self-concept of student athletes had a positive effect on their school life adaptation. These results reflect that the higher the physical self-concept of student athletes, the better their adjustment to class and rules and their relationship with teachers and classmates [29]. In other words, because student athletes understand their body, psychological factors such as self-esteem and sense of achievement are high [28]. This study’s finding regarding the effects of positive psychological factors is identical to that of a previous study [21] that such positive psychological factors had positive effects on school life.

Male student-athletes scored higher in TRE and SRU, whereas female athletes performed better in SCL and SRE. This gender difference may be based on endocrinological factors [30], with hormone secretion increasing under greater physical activity [31]. Regardless of the mechanism, we can conclude that boys are better at adjusting to rules and the regimented elements of school life, while girls are better at adjusting to classes and social relationships with their peers. This observation in female student-athletes corroborates results indicating that they have higher academic success [32]. However, this is coupled with lower athletic identity than boys, perhaps explaining why girls abandon sports at a 13.4% higher rate than abandonment rates among boys [33].

Based on these results, we recommend that parents and other mentors highlight the excellent performance of student-athletes to the students themselves. Understanding their own achievement should lead to the formation of beneficial relationships with authority figures, including teachers. A previous study found that female student-athletes with higher HP scores are likely to have better peer relationships, similar to a study on health concerns of middle school girls [34]. Notably, female students are more likely to discuss physical health concerns such as menstruation with their friends than their parents or managers [35,36,37]. Therefore, it may be particularly important for female students to have opportunities for peer engagement on health matters, in addition to counseling with their managers. Here, we also demonstrated that PA and HP affected school adjustment among female student-athletes more than SCO, perhaps because parents of girls are more concerned about their children’s well-being than higher performance [38].

Student-athletes who have not won awards exhibit a positive correlation between higher PA and most school-adjustment variables (excluding SRE). In contrast, among students who have won awards, higher SCO is associated with SCL and TRE. These results are consistent with previous research [39] showing that people tend to continue participating in activities that produce satisfactory outcomes. Through winning awards, the student-athletes consider themselves competent in sports, and this feeling of achievement appears to benefit their school adjustment. Therefore, we recommend encouraging appreciation of physical activity rather than sporting competence among students without award-winning experience, especially because steady exercise is rewarding even without a physical token of victory [40]. Our suggestion finds support in data showing that participation rates increase with greater awareness of physical activity’s benefits [41]. Mentors of student-athletes should aim to increase successful school adjustment through providing emphasizing such benefits, rather than blaming students for lack of awards.

Despite these encouraging findings, our study has several limitations. First, participants did it voluntarily, but a sample of convenience samples was used, and the participants were limited to secondary students. Future studies should therefore investigate the observed differences among elementary, secondary, and university student-athletes. Second, because student-athletes have varied motivations [42] that differentially influences burnout [43], we recommend that researchers examine such motivations for engaging in sports. Third, we only examined the potential mediating influence of two variables: sex and award-winning career. Future studies should also examine factors such as sporting-event types and player careers [44] to obtain a better understanding of factors that affect the academic achievement of student-athletes.

## 5. Conclusions

Analysis of the correlation between physical self-concept and school life adaptation showed that the high physical self-concept of student athletes had a positive effect on their school life adaptation. These results reflect that the higher the physical self-concept of student athletes, the better their adjustment to class and rules and their relationship with teachers and classmates [41]. In other words, because student athletes understand their body, psychological factors such as self-esteem and sense of achievement are high [42]. This study’s finding regarding the effects of positive psychological factors is identical to that of a previous study [28] that such positive psychological factors had positive effects on school life. Our results confirmed that student-athletes with higher physical self-concept are more likely to be successful in school adjustment. In particular, while students of both genders were better adjusted when PA scores were high, boys had improved adjustment with higher SCO, and girls, with higher HP. Thus, coaches and other mentors of student-athletes should develop management plans that build confidence for sports in a gender-specific manner. In addition, we found that SCO had greater influence on adjustment for student-athletes with award-winning careers, while PA was more influential for those without awards. These outcomes indicate that coaches should emphasize factors such as PA among non-award-winning students to better encourage successful school adjustment.

## Figures and Tables

**Table 1:** Results of Exploratory and Confirmatory Factor Analyses (EFA, CFA) for Physical Self-Concept.

Items	EFA			CFA		
	1 ^1^	2 ^2^	3 ^3^	1	2	3
SCO 2	0.817			0.778		
SCO 1	0.797			0.740		
SCO 3	0.763			0.701		
SCO 4	0.740			0.839		
SCO 5	0.634			0.739		
PA 3		0.791			0.749	
PA 1		0.782			0.778	
PA 4		0.756			0.721	
PA 2		0.700			0.747	
PA 5		0.576			0.654	
HP 2			0.882			0.576
HP 3			0.736			0.875
Cronbach’s α	0.873	0.846	0.850	NFI = 0.920		
Eigenvalue	5.786	1.265	1.014	TLI = 0.914		
Variance (%)	48.217	10.538	8.452	CFI = 0.934		
Variance accumulated (%)	48.217	58.755	67.208	RMR = 0.039		
KMO = 0.904, χ^2^ = 3421.509, df = 66, *p* < 0.001				CMIN = 275.517, DF = 51, *p* < 0.000		

^1^ Sports Competence (SCO), ^2^ Physical Activity (PA), ^3^ Health Promotion (HP).

**Table 2:** Results of Exploratory and Confirmatory Factor Analysis for School Adjustment Scale.

Items	Exploratory Factor Analysis				Confirmatory Factor Analysis			
	1 ^1^	2 ^2^	3 ^3^	4 ^4^	1	2	3	4
SCL 4	0.839				0.851			
SCL 2	0.780				0.772			
SCL 5	0.722				0.712			
SCL 6	0.690				0.750			
SCL 1	0.681				0.777			
SCL 3	0.635				0.588			
TRE 4		0.742				0.741		
TRE 5		0.723				0.808		
TRE 3		0.722				0.752		
TRE 6		0.712				0.732		
TRE 1		0.635				0.690		
TRE 2		0.556				0.435		
SRE 4			0.788				0.792	
SRE 2			0.769				0.835	
SRE 1			0.709				0.748	
SRE 5			0.678				0.606	
SRE 3			0.623				0.479	
SRU 3				0.702				0.758
SRU 7				0.689				0.743
SRU 4				0.689				0.791
SRU 2				0.611				0.673
SRU 6				0.610				0.721
SRU 1				0.605				0.596
Cronbach’s α	0.839	0.806	0.792	0.872	NFI = 0.872			
Eigen value	8.291	2.120	1.812	1.445	TLI = 0.890			
Variance (%)	36.049	9.220	7.876	6.284	CFI = 0.903			
Variance accumulated (%)	36.049	45.269	53.145	59.429	RMR = 0.039			
KMO = 0.920, χ^2^ = 6480.524, df = 253, *p* < 0.001					CMIN = 838.429, DF = 224, *p* < 0.000			

^1^ School Class (SCL), ^2^ Teacher Relationship (TRE), ^3^ Schoolmates Relationship (SRE), ^4^ School Rules (SRU).

**Table 3:** Demographic Characteristics and Questionnaire Scores by Variables (*n* = 589).

Variable	N	%	Variable	Mean (SD)
**Sex**			**Physical self-concept**	
Male	372	63.2%	SCO	3.84 (.727)
Female	217	36.8%	HP	4.06 (0.686)
**School**			PA	4.36 (0.618)
Middle	278	47.2%	**School adjustment**	
High	311	52.8%	SCL	3.93 (0.621)
**Award-winning career**			TRE	3.99 (0.641)
Yes	260	44.1%	SRE	4.00 (0.553)
No	329	55.9%	SRU	3.56 (0.705)

**Table 4:** Comparison of Mean between Physical Self-Content and School Adjustment.

Independent Variable	Dependent Variable	Mean Square	Standard Variation	F	Significance
SCO ^1^	SCL ^4^	3.21	0.411	7.813	0.001
	TRE ^5^	3.67	0.283	12.988	0.001
	SRE ^6^	2.84	0.335	8.489	0.001
	SRU ^7^	2.09	0.249	8.391	0.001
HP ^2^	SCL ^4^	2.74	0.474	5.790	0.001
	TRE ^5^	6.37	0.325	19.636	0.001
	SRE ^6^	6.04	0.353	17.130	0.001
	SRU ^7^	3.84	0.269	14.252	0.001
PA ^3^	SCL ^4^	3.64	0.403	9.049	0.001
	TRE ^5^	3.50	0.294	11.946	0.001
	SRE ^6^	3.01	0.334	9.051	0.001
	SRU ^7^	2.33	0.246	9.503	0.001

^1^ Sports Competence (SCO), ^2^ Health Promotion (HP), ^3^ Physical Activity (PA), ^4^ School Class (SCL), ^5^ Teacher Relationship (TRE), ^6^ Schoolmates Relationship (SRE), ^7^ School Rules (SRU).

**Table 5:** Comparison of Means between Physical Self-Content and School Adjustment.

Independent Variable	1	2	3	4	5	6	7
1. SCO ^1^	1						
2. HP ^2^	0.423 ***	1					
3. PA ^3^	0.643 ***	0.499 ***	1				
4. SCL ^4^	0.432 ***	0.224 ***	0.421 ***	1			
5. TRE ^5^	0.525 ***	0.407 ***	0.488 ***	0.531 ***	1		
6. SRE ^6^	0.427 ***	0.377 ***	0.413 ***	0.416 ***	0.500 ***	1	
7. SRU ^7^	0.400 ***	0.350 ***	0.436 ***	0.483 ***	0.496 ***	0.368 ***	1
*** *p* < 0.001							

^1^ Sports Competence (SCO), ^2^ Health Promotion (HP), ^3^ Physical Activity (PA), ^4^ School Class (SCL), ^5^ Teacher Relationship (TRE), ^6^ Schoolmates Relationship (SRE), ^7^ School Rules (SRU).

**Table 6:** Linear Regression of Physical Self-Concept and School Adjustment According to Sex (n = 589).

Dependent Variable	Independent Variable	Male				Female		
		β-Coefficient a	SE	*p*	β-Coefficient a		SE	*p*
SCL ^4^	SCO ^1^	0.266	0.059	0.000	0.296		0.079	0.000
	HP ^2^	0.264	0.071	0.000	0.233		0.078	0.005
	PA ^3^	−0.041	0.064	0.464	0.004		0.077	0.959
TRE ^5^	SCO ^1^	0.366	0.046	0.000	0.243		0.067	0.001
	HP ^2^	0.207	0.056	0.001	0.208		0.066	0.006
	PA ^3^	0.100	0.050	0.051	0.282		0.065	0.000
SRE ^6^	SCO ^1^	0.267	0.051	0.000	0.179		0.074	0.031
	HP ^2^	0.215	0.062	0.001	0.129		0.073	0.116
	PA ^3^	0.128	0.056	0.017	0.278		0.072	0.000
SRU ^7^	SCO ^1^	0.176	0.044	0.003	0.145		0.066	0.077
	HP ^2^	0.235	0.053	0.000	0.230		0.065	0.005
	PA ^3^	0.124	0.048	0.027	0.230		0.064	0.001

^1^ Sports Competence (SCO), ^2^ Health Promotion (HP), ^3^ Physical Activity (PA), ^4^ School Class (SCL), ^5^ Teacher Relationship (TRE), ^6^ Schoolmates Relationship (SRE), ^7^ School Rules (SRU).

**Table 7:** Linear Regression of Physical Self-Concept and School Adjustment According to Award (n = 589).

Dependent Variable	Independent Variable	Granular Experience NO			Granular Experience YES		
		β-Coefficient a	SE	*p*	β-Coefficient a	SE	*p*
SCL ^4^	SCO ^1^	0.256	0.069	0.001	0.273	0.065	0.000
	HP ^2^	0.267	0.083	0.001	0.262	0.066	0.000
	PA ^3^	−0.050	0.075	0.462	0.001	0.064	0.988
TRE ^5^	SCO ^1^	0.267	0.055	0.000	0.352	0.053	0.000
	HP ^2^	0.299	0.067	0.000	0.128	0.054	0.041
	PA ^3^	0.165	0.060	0.006	0.166	0.053	0.002
SRE ^6^	SCO ^1^	0.254	0.062	0.001	0.212	0.058	0.001
	HP ^2^	0.185	0.075	0.022	0.163	0.060	0.011
	PA ^3^	0.128	0.067	0.055	0.243	0.058	0.000
SRU ^7^	SCO ^1^	0.179	0.056	0.018	0.167	0.049	0.008
	HP ^2^	0.285	0.068	0.001	0.229	0.050	0.000
	PA ^3^	0.073	0.061	0.281	0.213	0.048	0.000

^1^ Sports Competence (SCO), ^2^ Health Promotion (HP), ^3^ Physical Activity (PA), ^4^ School Class (SCL), ^5^ Teacher Relationship (TRE), ^6^ Schoolmates Relationship (SRE), ^7^ School Rules (SRU).
